# SWCNT–porphyrin nano-hybrids selectively activated by ultrasound: an interesting model for sonodynamic applications†

**DOI:** 10.1039/d0ra03944f

**Published:** 2020-06-08

**Authors:** Federica Bosca, Ingrid Corazzari, Federica Foglietta, Roberto Canaparo, Gianni Durando, Linda Pastero, Silvia Arpicco, Franco Dosio, Daniele Zonari, Giancarlo Cravotto, Silvia Tagliapietra, Loredana Serpe, Francesco Turci, Alessandro Barge

**Affiliations:** Department of Drug Science and Technology, University of Turin Turin Italy alessandro.barge@unito.it; Department of Chemistry, University of Turin Turin Italy; National Institute of Metrological Research (INRIM) Turin Italy; Department of Earth Sciences, University of Turin Turin Italy; “G. Scansetti” Interdepartmental Centre, University of Turin Via P. Giuria 9 10125 Turin Italy

## Abstract

Sonodynamic therapy (SDT) is an innovative anticancer approach, based on the excitation of a given molecule (usually a porphyrin) by inertial acoustic cavitation that leads to cell death *via* the production of reactive oxygen species (ROS). This study aims to prepare and characterize nanosystems based on porphyrin grafted carbon nanotubes (SWCNTs), to understand some aspects of the mechanisms behind the SDT phenomenon. Three different porphyrins have been covalently linked to SWCNTs using either Diels–Alder or 1,3-dipolar cycloadditions. ROS production and cell viability have been evaluated upon ultrasound irradiation. Despite the low porphyrin content linked on the SWCNT, these systems have shown high ROS production and high tumour-cell-killing ability. The existence of a PET (photoinduced electron transfer)-like process would appear to be able to explain these observations. Moreover, the demonstrated ability to absorb light limits the impact of side effects due to light-excitation.

## Abstract 2

Sonodynamic therapy (SDT) is an innovative anticancer approach, based on the excitation of a given molecule (usually a porphyrin) by inertial acoustic cavitation that leads to cell death *via* the production of reactive oxygen species (ROS).

## Graphical abstract

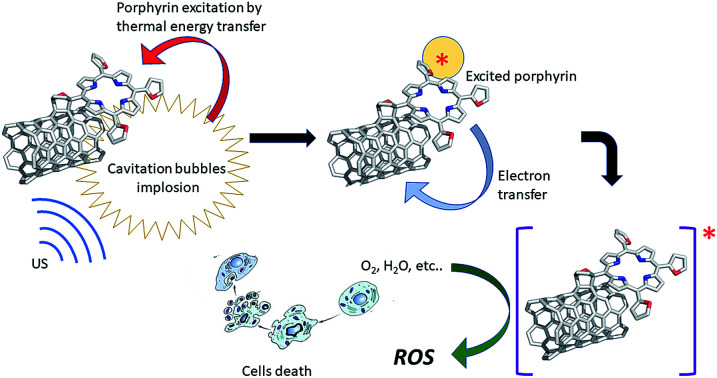

## Introduction

Sonodynamic therapy (SDT) was introduced as an innovative anticancer approach in 1989.^1,2^ Three main players are involved in this treatment, namely: (i) low-intensity ultrasound (US) used as the energy source, (ii) a chemical sonosensitizer that is activated by US induces the production of reactive oxygen species (ROS), and (iii) the ROS themselves, which damage and kill target cells.^3^ The molecular mechanism of SDT is still unknown, although several hypotheses have been proposed.^4^ Sonoluminescence as well as sonochemical and physical effects^1^ (namely high pressure and temperature), caused by the implosion of cavitation gas bubbles (namely inertial cavitation), are thought to be mainly involved. Sonoluminescence is a process, invoked in a controversial way,^5,6^ in which gaseous species dissolved in solution are excited by acoustic cavitation and become able to emit light. The light emitted would excite sonosensitizer molecules (usually a porphyrin ring) which, in turn, induces the generation of ROS (including singlet oxygen, and hydroxyl radicals). On the other hand, the sonosensitizer excitation could be directly produced by the high punctual temperature and pressure conditions induced by cavitation, following a thermal energy transfer mechanism. Moreover, direct radical production from water or from the sonosensitizer itself could also be implied.^7–9^ Porphyrin derivatives, which are non-toxic to cell culture *per se*^10^ are considered as the first-generation sensitizers and extensively used as model compounds in photo- and sono-dynamic treatment.^11,12^ However, in the clinical practice prolonged skin photosensitivity after systemic administration of porphyrins is observed.^13^ This side-effect represents one of the major shortcoming in the clinical use of such sensitizers.^14^ To overcome this problem it is possible to select sonosensitizer with different structure, or to select molecules able to be excited only by US. Another strategy envisages the enhancement of the sonosensitizer ability to generate ROS. This approach allows to reduce the dose and, consequently, the sonosensitizer concentration in the skin. Furthermore, the sonosensitizer may also be made tissue selective by conjugating it to a specific vector or including it in targeted nanoparticles. To conjugate sonosensitizer to vector, more than one strategy can be simultaneously followed. Single walled carbon nanotubes (SWCNT), are one of the most promising materials in nanomedicine^15,16^ and they represent an ideal model to build a new kind of porphyrin based sonosensitizer (porphyrin–SWCNT conjugated). SWCNT are known for their use in drug delivery and targeted drug delivery,^17,18^ but they are also known for their peculiar dimensional features (micrometric dimension along *z* axis, and stiffness) and electronic properties.^19–21^ Three main advantages are expected from the porphyrin–SWCNT nanohybrid: (i) as described by Baskaran and co-workers,^22^ the stiffness and micrometric size of SWCNT bundle favour the inertial acoustic cavitation to occur in the proximity of the carbon surface where the sonosensitizing agent is linked, (ii) the exceptional electron mobility of SWCNT^23^ assists the electron transfer between covalently bonded molecules and the nanotube, imparting a US-harvesting antennae behaviour^20,22,24–26^ and (iii) SWCNT allows a facile chemical grafting^16^ of porphyrin derivatives onto the carbon surface and allow further functionalization with target agents.^15,17,27,28^ In this study three different porphyrin–SWCNT nanohybrids were synthesized and studied in *in vitro* sonodynamic applications.

## Results and discussion

**Fig. 1:**
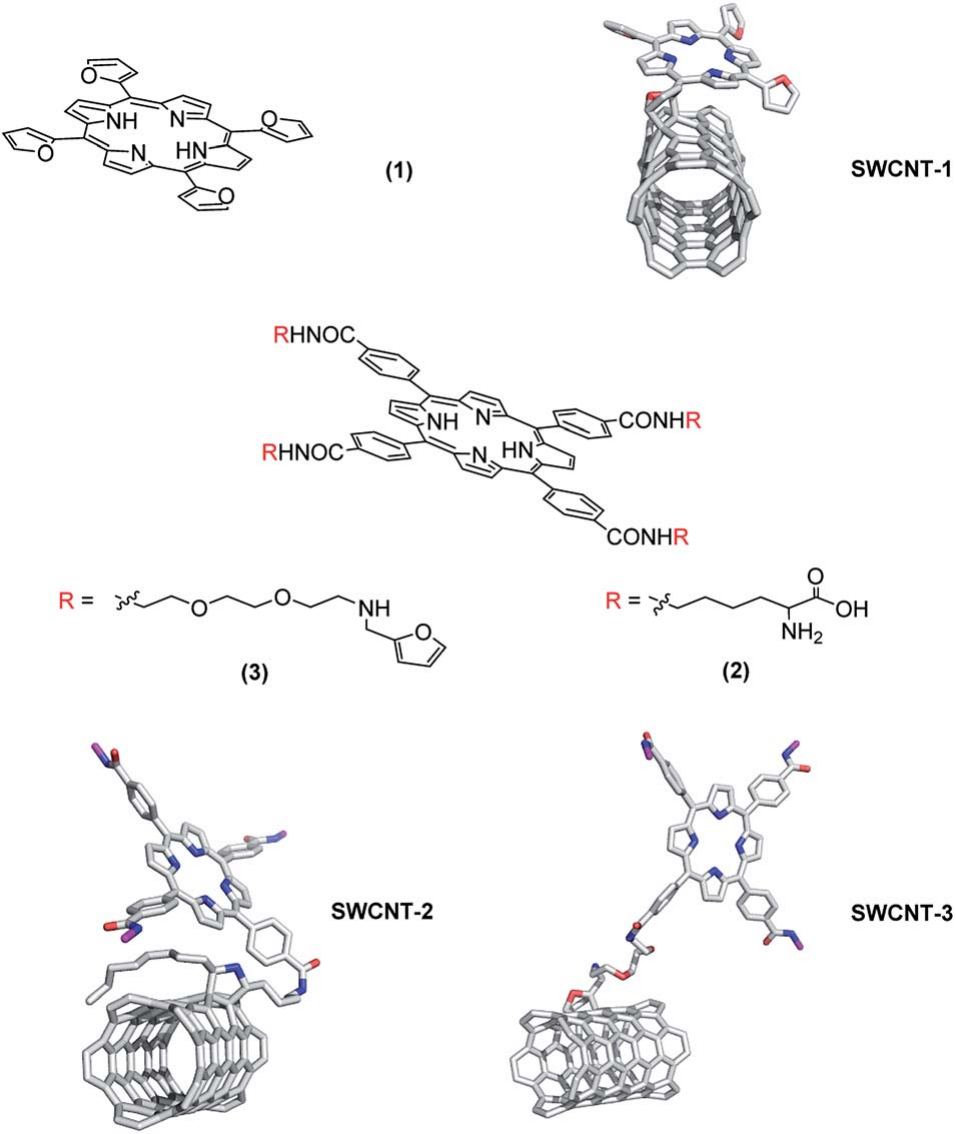
Structures of synthesized porphyrins and 3D pictures of porphyrins SWCNTs grafted.

To assess if the ability in amplifying/facilitating the inertial cavitation conditions and the electron transfer phenomenon from porphyrin to SWCNT may increase toxic effect on cancer cells, we conjugated the SWCNT surface with three *ad hoc* tailored porphyrin derivatives. The three nanohybrid systems (namely SWCNT–1, -SWCNT–2 and SWCNT–3), obtained by grafting these porphyrins onto SWCNT surface (Fig. 1), differ in linking modality and consequently in spatial relative disposition. Porphyrins derivatives 1 and 3 were obtained as previously reported,^29,30^ whereas derivative 2 was synthesized *via* condensation of *meso*-tetrakis-(carboxyphenyl)-porphyrin with α-Boc-lysine *t*-butylester in presence of ethyl-diisopropylcarbodiimide (EDC) and 4-*N*,*N*-dimethylaminopyridine (DMAP).

Porphyrins 1 and 3 expose furanyl terminal groups, which are appropriate for Diels–Alder (DA) cycloadditions. Porphyrin 2 was synthetized to employ the α-aminocarboxylic moiety as precursor of azomethine ylides, which is exploitable in 1,3-dipolar cycloadditions. All grafting reactions with porphyrin 1, 2 and 3, were performed by mono-modal microwave enhanced cycloaddition. The functionalization degree of these three hybrid materials was assessed by thermogravimetric analysis (Fig. S7, ESI†).

The thermal degradation of CNT–porphyrin conjugates was followed in the temperature range 270–700 °C. Three regions were detected, namely at 25–160 °C (region I) and 160–270 °C (region II) the desorption of molecular water and traces of solvent deriving from the synthesis (*N*,*N*-dimethylformamide, DMF) accounts for a minor weight loss (<3%). The weight loss at this temperature (270 °C) was used as reference point (offset) to calculate the amount of porphyrin of each conjugate. The amount of porphyrin was quantified by subtracting the offset of each curve at 270 °C from the weight loss at 700 °C, in which the thermal degradation of porphyrin is completed. The percentage of porphyrin per mass unit of conjugate (SWCNT + porphyrin) introduced by the functionalization process in SWCNT–1, SWCNT–2 and SWCNT–3 was 6.7% w/w, 8.0% w/w and 7.0% w/w, respectively. To obtain the functionalization degree (*i.e.*, the molar concentration of porphyrin per mass unit of conjugate) the wt% were converted by using the molar mass of each porphyrin. The functionalization degree of SWCNT–1, SWCNT–2, and SWCNT–3 reported as μmol of porphyrin per mg of sample was calculated as follows: functionalization degree = (*Δ*_700–270 °C_ (mass loss%)/MW porphyrin) × 10^3^. This result (Table 1) evidenced that similar functionalization degrees were obtained in all cases, highlighting that the different grafting reactions, as well as the distance between furanyl moiety and porphyrin core, do not significantly affect the grafting yield, which ranged in all cases form 4.3 × 10^−2^ to 1.2 × 10^−1^ μmol of porphyrin per mg of sample.

**Table 1:** The functionalization degree of SWCNT–1, SWCNT–2, and SWCNT–3 reported as μmol of porphyrin per mg of sample was calculated as follows: functionalization degree = (*Δ*_700–270 °C_ (mass loss%)/MW porph) × 10^3^

Nano-hybrid	Porphyrin MW (mg mmol^−1^)	Porphyrin wt%	Functionalization degree (μmol mg^−1^)
SWCNT–1	574	6.7%	1.2 × 10^−1^
SWCNT–2	1411	8.0%	5.7 × 10^−2^
SWCNT–3	1634	7.0%	4.3 × 10^−2^

With the purpose to highlight a possible role of porphyrin linking modality, the toxic effect of these hybrid nanosystems on tumour cells was then evaluated. To proceed with cellular studies, it was necessary to stabilize the aqueous suspension of the modified SWCNT, reducing the aggregation phenomena. The three SWCNT–porphyrin hybrids were wrapped with PEG (PEG = polyethylene glycol) chains by using mPEG-DSPE (mPEG-DSPE = 1,2-distearoyl-*sn*-glycero-3-phosphoethanolamine-*N*-[amino(polyethylene glycol)-2000] ammonium salt) under mild conditions.^31^ The attachment of PEG was confirmed by thermogravimetric analysis (Fig. S6, ESI†), which reveals the presence of one mPEG-DSPE chain each 104 carbon atoms (weight loss of 69%, 250 μmol per g of SWCNT).

The hydrophobic lipid chains of the DSPE moiety interact with the lipophilic carbon surface, decorating the SWCNT with a PEG sunburst pattern and increasing the stability of SWCNT aqueous suspension up to 30 days (Fig. S5, ESI†). A human colorectal cancer cell line (HT-29), a well-known cellular model, was then selected for *in vitro* tests. Cells were incubated with a suspension of SWCNT–porphyrin–PEG (porphyrin: 1, 2 or 3) or SWCNT–PEG (negative control) and exposed to US irradiation. The results were compared to those obtained employing light irradiation (since porphyrin is activable also by light), following previously published procedures.^11,21,24^ Cell proliferation at 24 and 48 hours after each treatment was assessed (Fig. 3). The incubation with SWCNT–1–PEG, SWCNT–2-PEG, and SWCNT–3–PEG under US irradiation elicited a significant reduction of the cellular growth over time (up to 75%). Cell viability upon US irradiation was not affected by the mode of conjugation, since all the three different grafting procedures gave very similar and very low substitution degree and the differences shown in Fig. 3, event after 48 h of incubation, were not statistically relevant. Light irradiation of functionalized SWCNT and non-functionalized SWCNT under both light- and US-irradiation did not induce any significant alteration in cell proliferation.

It is known that not conjugated porphyrins having similar chemical structure to those used in this study are able to generate ROS when light irradiated and kill cells^32–34^ However, in our experimental conditions, the lack of effect with light irradiation is likely due to the absorption of most of the impinging photons by the large black SWCNT surface, which, combined with the modest amount of porphyrin grafted, prevents photoexcitation. To clarify the chemical mechanism at the basis of the observed cell effect, reactive oxygen species (ROS) generation by porphyrin loaded SWCNT hybrid was assessed by employing, terephthalate assay.^35^ US irradiated porphyrin promotes ROS production with consequent generation of hydroxyl radicals which convert terephthalate into its fluorescent derivative 2-hydroxyterephthalate (TA-OH). SWCNT–1 in DMSO : water (1 : 3) suspension was subjected to US irradiation in the dark. The experiments were carried out under two different US irradiation conditions: 1.866 MHz and 22 kHz. The former was selected in order to match the experimental condition used in cell treatments and the latter to test our system in well-studied ROS production conditions.^2^ ROS production of SWCNT–1 was compared with pristine tube (SWCNT) under the same experimental conditions adopted in cell experiments. To take into account ROS production *via* water sonolysis, an aqueous solution of TA was irradiated under the same conditions without SWCNT (blank + US). All the results were also compared to a solution not subjected to sonication (blank), to consider TA autoxidation. Under both US frequencies, the ROS generated by SWCNT–1 were significantly higher than those observed with pristine SWCNT (*p* < 0.05). Notably, pristine SWCNT did not induce ROS production more than blank solution, indicating that the radical generation by our hybrid nanomaterial relays on the occurrence of porphyrin bonded to SWCNT surface.

The results explain that cell death is a consequence of solely US treatment, whereas light irradiation does not provide any effect on cell vitality. This phenomenon could be ascribed to the low amount of porphyrin grafted on the tube surface, which is enough to promote a remarkable US-induced cellular toxicity, but insufficient to be excited by light (photons are mainly absorbed by SWCNT surface). SWCNT–porphyrin behaves as a selective US-activated hybrid nanosystem. The thermal energy, coming from inertial cavitation, is efficiently transferred to the few porphyrin units linked to the nanotube surface, causing molecule excitation, then, similarly, to what happens in a photo-induced electron transfer (PET) process, the electron transfer between excited porphyrin (electron-donator) and carbon nanotube aromatic surface (electron-acceptor) stabilises the separated-charge state, increasing the probability of molecular oxygen to react with the excited system and facilitating the ROS production *via* US excitation (Fig. 4).

This feature can be further highlighted by comparing cell toxicity induced by our SWCNT–porphyrin system with that induced by-decorated polymeric core–shell nanoparticle (PCNP), under the same experimental conditions.^24^ A 40-fold lower dose of porphyrin grafted on SWCNT is sufficient to induce a similar cell toxicity. So far, various porphyrin nanocarriers have been investigated due to their inherent ability to improve the triggering effect US has on the sonosensitizing agent.^36,37^ However, CNT-based nanocarriers shows unique properties, such as the photoinduced electron transfer, making them an intriguing multifunctional hybrid nanoplatform suitable for the development of the next generation of US responsive nanosystems. Moreover, the results reported herein are supported by the work of Yumita *et al.*^38,39^ highlighting the great potential of CNT-based nanosystems in exploiting the US-mediated ROS generation, representing the pivotal step of sonodynamic applications.

**Fig. 2:**
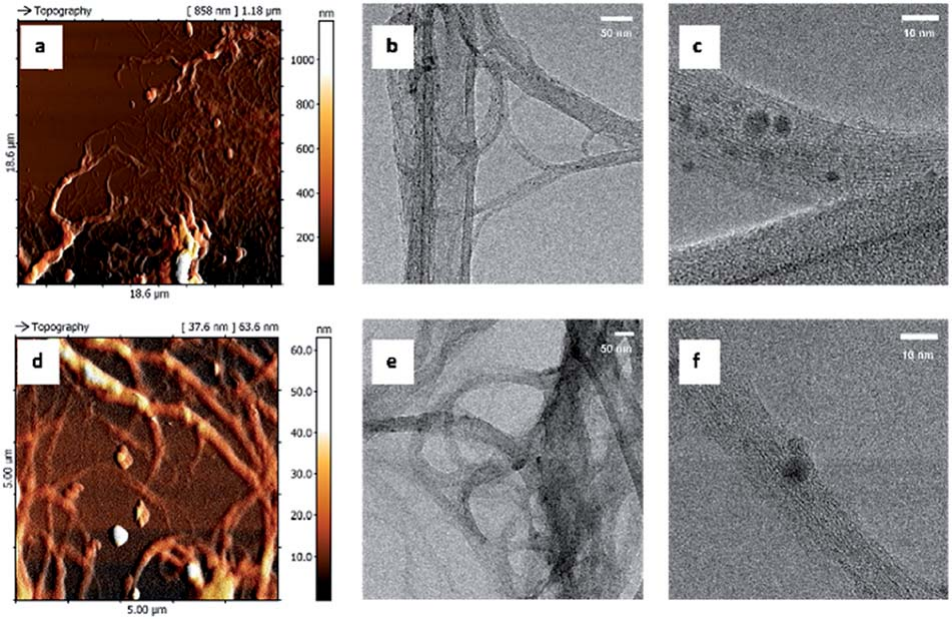
AFM and TEM images of SWCNT–1 (a–c) and pristine SWCNT (d–f).

Since the synthetic procedures share the same pressure and temperature conditions, same type of reaction mechanism (*i.e.* pericyclic reaction), produces very small amount of porphyrin grafted on SWCNT and, especially, the three nanosystems show very similar biological behaviour, the structural and morphological properties of these nanohybrids were assessed only on SWCNT–1, using several complementary physico-chemical characterization techniques. The results were compared with those obtained with pristine carbon nanotubes (SWCNT). Atomic force microscopy (AFM) and transmission electron microscopy (TEM) images (Fig. 2) were used to assess the nano-morphology of pristine and grafted SWCNT. High electron-dense areas (dark spots in panels c and f, in Fig. 2) are consistent with the presence of metal residues and amorphous carbon attached to SWCNT bundles.^23^ The smallest structures detected by AFM were composed of about ten single nanotubes, which were directly observed by HR-TEM (panel b and e) and showed a minimum width of 7 nm. No significant morphological modifications were induced by the grafting procedure.

**Fig. 3:**
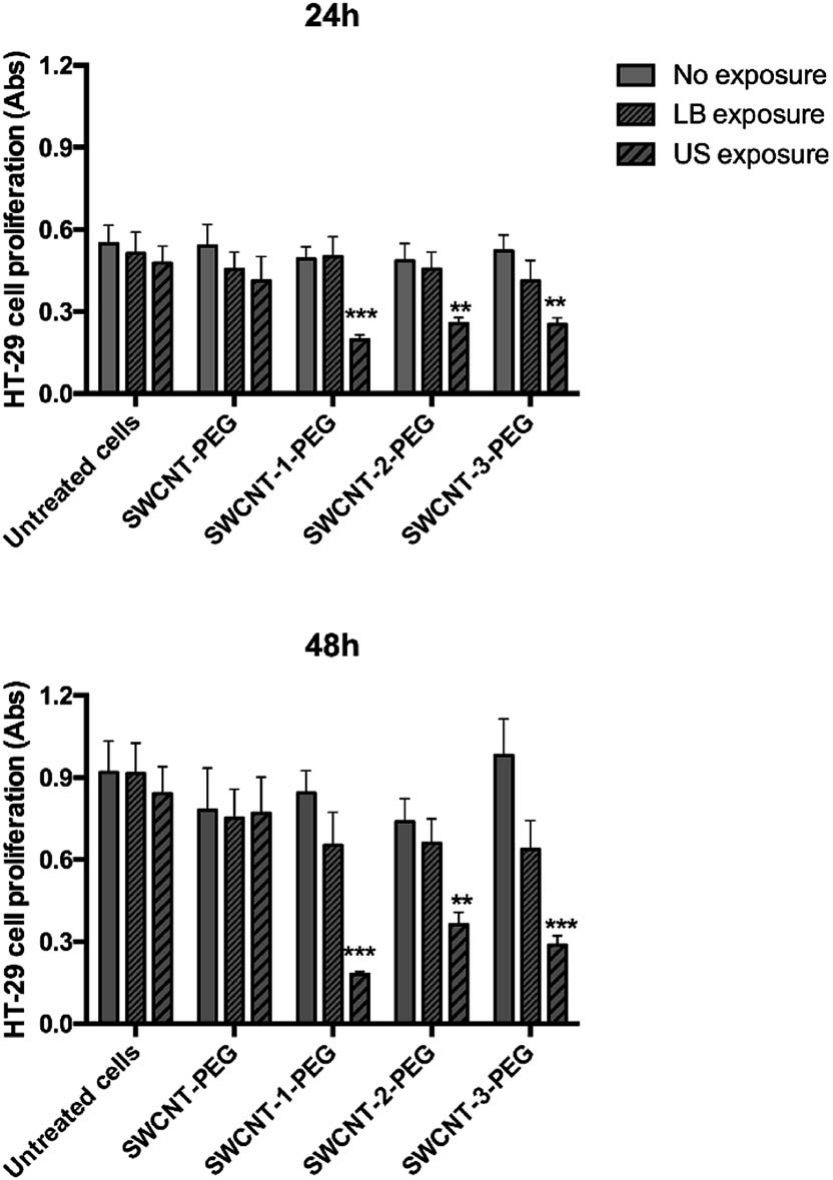
Effect of the exposure of porphyrin-loaded SWCNT to LB and US on HT-29 cell proliferation. HT-29 cells were exposed to porphyrin-loaded SWCNT–PEG (SWCNT–1–PEG; SWCNT–2–PEG and SWCNT–3–PEG, at 25μg ml^−1^) and SWCNT–PEG and then either LB (15 mW cm^−2^ for 5 minutes, 405 nm) or US (0.008 mJ cm^−2^ for 5 min, 1.866 MHz). Cell proliferation was evaluated after 24 and 48 h by WST-1 assay. Statistical significance between no exposure (full bars) and LB or US exposure (dashed bars): **p < 0.01, ***p < 0.001.

**Fig. 4:**
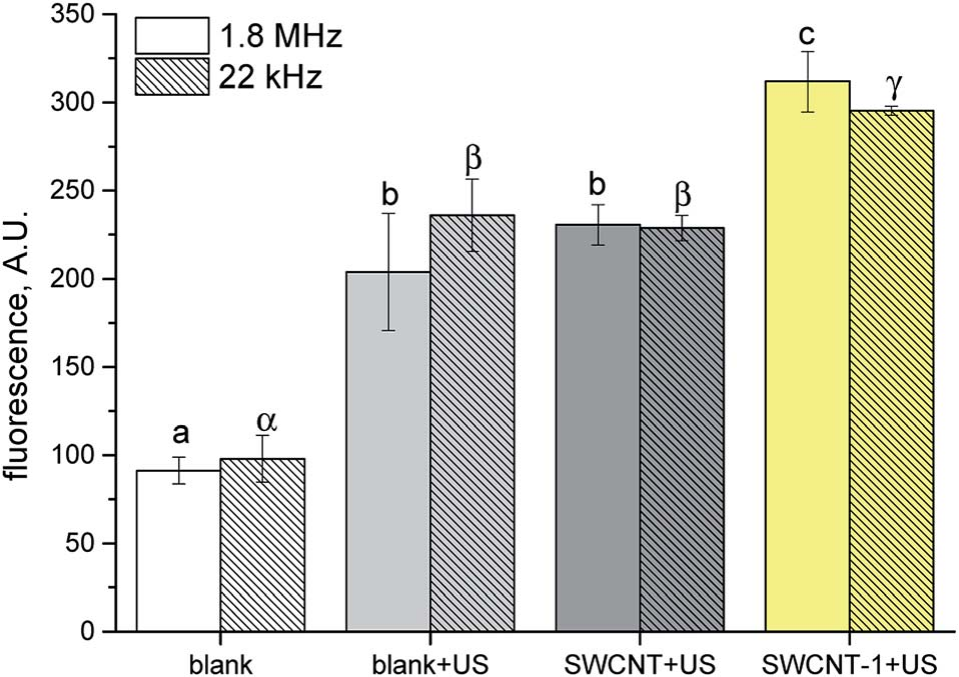
TA hydroxylation induced by SWCNT–1 sonicated at different frequencies (1.8 MHz and 22 kHz). Pristine and porphyrin-conjugated SWCNT (SWCNT and SWCNT–1, respectively) suspensions and TA only solution (blank) were sonicated (+US) for 5 min and 1 min with an ultrasound probe operated at 1.8 MHz and 22 kHz, respectively. Data are reported as mean ± standard deviation of triplicated experiments. Columns that do not share at least one letter are statistically different (ANOVA, Tukey test *p* < 0.05).

**Fig. 5:**
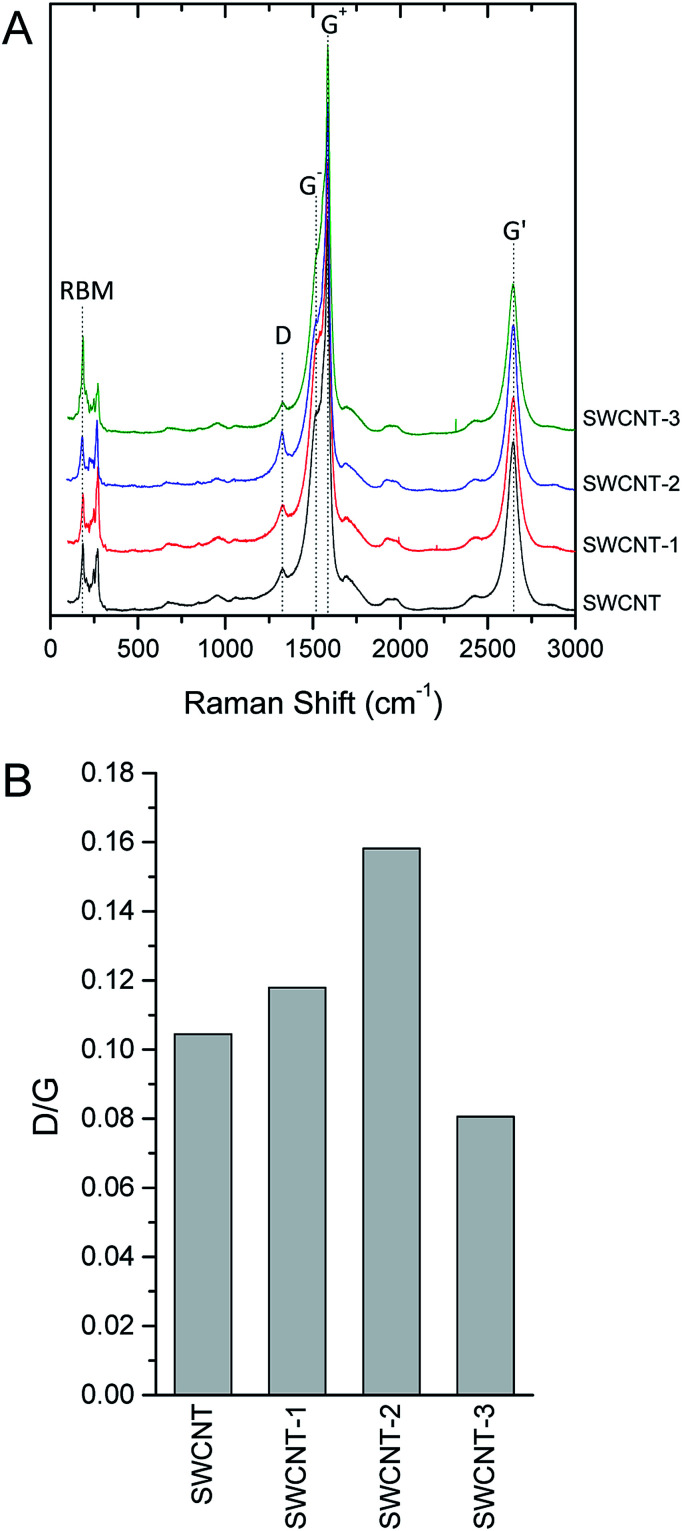
Representative Raman (*λ*_ex_ = 532 nm) spectra (A) and relative intensity of the D-band (B) of pristine (SWCNT) and variously conjugates nanohybrids (SWCNT–1, SWCNT–2 and SWCNT–3). Raman graphitic band, split in the two singularities (G^+^, for atomic displacements along the tube axis, and G^−^ for modes with atomic displacement along the circumferential direction), ring breathing modes (RBM), and disorder band (D) were observed in all spectra. For each spectrum, the relative intensity of the disorder band with respect to G^+^ band (*I*_D_/*I*_G_) was calculated.

As previously observed on reduced graphene oxide grafted with porphyrin,^21^ Raman spectroscopy confirmed that the functionalization process does not significantly modify SWCNT structure (Fig. 5). Raman spectroscopy of carbon nanotubes, and crystalline graphite in general, is characterized by two strong peaks centred at *ca.* 1580 cm^−1^ and 2700 cm^−1^ (G and G′ bands, respectively). The lowest frequency peak (G-band, after graphite) is a first-order Raman-allowed feature originating from the in-plane stretching of the C–C bond. The highest frequency peak (G′ band) is usually assigned to a second order two-phonon feature of crystalline graphite and, generally, of all sp^2^-carbon systems. In SWCNT, the rolled structure of the tube breaks the two-dimensional graphene symmetry and up to six high-energy tangential modes are allowed for the G-band. Those modes can be practically treated considering only two intense superimposed peaks labelled G^+^, for atomic displacements along the tube axis, and G^−^, for modes with atomic displacement along the circumferential direction. The G^+^ frequency is constant with diameter for metallic and semiconductor tubes, while G^−^ can be used to infer about the strain affecting CNT structure, including nanotube curvature. At lower Raman shift (*ca.* 1350 cm^−1^), a peak is observed in the presence of defects that discontinue the otherwise perfect sp^2^ graphite structure. This peak is conventionally named D-band, where D stands for defect or disorder. D-band in SWCNT is commonly used to discuss the disorder-induced features related to symmetry breaking of the sp^2^ configuration of graphene sheet. At Raman shift ranging from 160–280 cm^−1^ radial breathing mode (RBM) are usually observed for SWCNT. Fig. 5 A shows representative spectra obtained from bundles of SWCNT with a low aperture number objective and a 532 nm laser excitation source. Raman spectra were background subtracted with a second-order polynomial fitting baseline and normalized for the Raman intensity of the G band. As expected, the most prominent feature of pristine SWCNT was the G-band at *ca.* 1580 cm^−1^ showing the two G^+^ and G^−^ components at 1583 and 1516 cm^−1^, respectively, and the G′-band at 2645 cm^−1^. The sharpness and moderate downshift of the G^−^ band of the pristine SWCNT signals the semiconducting nature of the tube used in this work. The D-band is observed at 1326 cm^−1^. A qualitative comparison of the peak shapes and frequencies between pristine and conjugated SWCNT evidenced that the structural features of the tube were largely preserved during the porphyrin conjugation. Because absolute intensity measurement is a difficult task in Raman spectroscopy, the normalized intensity *I*_D_/*I*_G_ ratio was used to quantitatively describe the amount of disorder (Fig. 5B) eventually induced by conjugation. Bearing in mind that the D-band is not sensitive to all kind of defects, the relative low intensity of this band with respect to the G-band clearly indicates that the pristine SWCNT has a low amount of defective rings, *i.e.* sp^3^ carbon atoms, open-end tubes, and sidewall holes. To take into account that the laser source may easily overheat and damage the samples, thus altering the Raman intensity of D-band, a set of spectra were recorded on the same spot and tube bundles were observed to be stable under laser irradiation for minutes or even longer (data not shown). All the conjugates obtained showed a negligible increase of the relative intensity of the D-band with respect to G^+^-band (*I*_D_/*I*_G_ ratio), exhibiting ratios between 0.08 and 0.16. To comparative analyse this range of values, one should consider that a defective SWCNT might exhibit *I*_D_/*I*_G_ ratio > 1.6.^40^ The relative intensity and the downshift of G^−^-band is virtually the same for all samples, indicating that conjugation-induced strain did not significantly affect tube curvature. Furthermore, the functionalization did not induce shifts in the main features of RBM at 186 and 270 cm^−1^, further confirming the stability of tube diameter over conjugation. Overall, the Raman spectra of the pristine and the variously characterized SWCNTs were superimposable and the structural integrity of tubes after functionalization further confirmed.

## Experimental

### Materials and methods

All reagents and solvents were purchased from Sigma-Aldrich and used without further purification, *meso*-tetrakis(4-carboxyphenyl)porphyrin was purchased from Frontier Scientific, Pure HiPco single walled carbon nanotube was purchased from Unidym. MW assisted reactions were carried out in a monomodal reactor Monowave 300 (Anton Paar) equipped with a monitoring system of temperature, pressure and power input. The CombiFlash RfsTeledyne (ISCO) purification system was used to purify all crude reactions on silica gel. Analytical and preparative HPLC-MS was performed using a FractionLink autopurification system (Waters) equipped with a 2996 photo diode array detector and Micromass ZQ detector (ESCI hybrid ionization source). NMR spectra were recorded on an Avance 300 or on an Avance 600 spectrometers (Bruker) operating respectively at 7 T and 14 T, dissolving samples in proper deuterated solvent. Chemical shifts were referenced per residual solvent signals. MS spectra were carried out using electrospray ionization (ESI) or by atmospheric pressure chemical ionization (APCI), in positive ion mode, on a Micromass ZQ spectrometer (Waters).

Thermogravimetric analysis: TGA analyses were carried out under dynamic nitrogen atmosphere (35 ml min^−1^) by a Pyris 1 ultra-micro-balance; the gases evolved during the heating ramp were continuously monitored by a Spectrum 100 FTIR spectroscope (Perkin Elmer). The gas evolved during the heating ramp was piped (gas flow 65 ml min^−1^) *via* pressurized heated (280 °C) transfer line (Redshift S.r.l.) and analysed continuously by the FTIR (Spectrum 100, Perkin Elmer), equipped with a thermostated (280 °C) conventional gas cell. The spectra were acquired in the 4000–600 cm^−1^ wavenumber range with a resolution of 0.4 cm^−1^ and analysed with the Spectrum software (Perkin Elmer). Temperature-resolved infrared profiles of the identified volatiles evolved from samples were obtained from the intensity of a representative peak of the investigated species.

TEM analyses: the morphology of the samples was investigated in the nanometric range by means of a JEOL 3010-UHR transmission electron microscope (TEM) equipped with a LaB_6_ filament operated at 300 kV, beam current = 114 μA and equipped with a 2k × 2k pixels Gatan US1000 CCD camera. Micro-Raman spectroscopy: the laser beam of a confocal Raman microscope (Horiba Jobin-Yvon HR800 and Olympus BX41 microscope) has been focused on a 1 μm × 1 μm wide portion of the SC. A polarized solid-state Nd laser operating at a wavelength of 532.11 nm and power of 80 mW and a CCD air-cooled detector operating at −70 °C were used. Calibration of the instruments was performed by measuring the Stokes and anti-Stokes bands of the Si band at 520.7 cm^−1^. Samples were placed on a polished stainless-steel slide and a 50× objective delivering a power of *ca*. 15 mW on the sample was used. Spectra were acquired with a spectral resolution of *ca.* 2 cm^−1^ and an integration time spanning from 100 to 400 seconds.

Atomic force microscopy (AFM): AFM height profile measurements were performed using a DME SPM Microscope (DME Igloo) equipped with a DS95-50E scanner (scan volume 50 × 50 × 5 μm). The data were acquired using MikroMasch Ultrasharp NSC16/Si_3_N_4_ Cr–Au back-coated cantilevers with typical resonance frequency 190 kHz, force constant 45 N m^−1^, tip radius lower than 35 nm and full tip cone angle 40°. Due to the softness of the samples, all measurements were performed in alternated contact mode.

Light exposure apparatus: a custom system for LB exposure with fixed source-tube positioning and light diffuser all-around cell culture was used. The light-emitting source of the system is based on InGaN light-emitting diodes (Cree Inc.) with 20 mW max radiant power at a central wavelength of 405 nm. The energy fluency rates of the light radiation were adjusted to 15 mW cm^−2^ for 5 min, measured using Actinic UV-meter (Jelosil).

US exposure apparatus: the US field was generated by a plane wave transducer in continuous wave at *f*_0_ = 1.866 MHz connected to a power amplifier (Type AR 100 A250A; Amplifier Research, Souderton, USA) and a function generator (Type 33250; Agilent). A mechanical adaptor was built to connect the 1 cm diameter polystyrene tube containing the cells suspended in PBS. When filled with ultrapure water, the adaptor creates highly reproducible measurement conditions at a fixed cell tube distance from the transducer.

Cell viability was evaluated by absorbance measurements (450 and 620 nm as reference wavelength) on a microplate reader (Asys UV340, Biochrom).

### Synthetic procedures

Compound 1 and 3 were synthesized accordingly with reported procedures.^30^ Whereas the procedure to obtain compound 2 is here reported.

#### Synthesis of compound (1)

Compound (1) was synthesized accordingly to previously described procedure,^29^ developed by authors, in 2% global yield.

^1^H NMR (300 MHz, THF-d8, *δ* 1.73 ppm) *δ* 9.18 (s, 8H), 8.25 (br s, 4H), 7.39 (br s, 4H), 7.08 (br s, 4H), −2.55 (s, 2H); APCI-MS^+^: *m*/*z* calcd for C_36_H_22_N_4_O_4_, 574.16; found 575.48 [M + H^+^].

#### Synthesis of compound (a) (CBzLys-*t*-BuO): *tert*-butyl 2-amino-6-(((benzyloxy)carbonyl)-amino)-hexanoate

Lys-CBz (10 g, 35.7 mmol) was dissolved in terz-butyl acetate (170 ml) and HClO_4_ (70% in water, 42 mmol, 3.6 ml) was slowly added. The mixture was stirred at RT for 18 h, then water (155 ml) was added. The two phases were separated and the aqueous layer was washed with ethyl acetate (2 × 50 ml). The organic layers were then combined and washed with a 5% NaHCO_3_ solution (2× 100 ml), dried over sodium sulphate and solvent was removed to obtain (a) in 94% yield (11.25 g). ^1^H NMR (300 MHz, DMSO-d_6_, *δ* 2.50 ppm) *δ* 7.32 (m, 5H), 7.19 (t, *J* = 5.42 Hz, 1H), 4.99 (s, 2H), 3.82 (t, *J* = 5.95 Hz, 1H), 3.00 (m, 2H), 1.72 (m, 2H), 1.50–1.15 (overlapped signals, 13H); ^13^C NMR (75 MHz, DMSO-d_6_, *δ* 39.5 ppm) *δ* 169.6, 156.8, 137.8, 129.0, 128.4, 128.3, 83.5, 80.2, 65.8, 52.9, 30.6, 29.4, 28.1, 22.0; ESI-MS^+^: *m*/*z* calcd for C_18_H_28_N_2_O_4_, 336.42; found 337.19 [M + H^+^].

#### Synthesis of compound (b) (CBzLysBoc-*t*Bu): *tert*-butyl 6-(((benzyloxy)carbonyl)-amino)-2-((*tert*-butoxycarbonyl)-amino) hexanoate

Compound (a) (1.0 g, 2.97 mmol) was added to 50 ml of CH_2_Cl_2_ and heated under reflux until complete dissolution. The solution was allowed to reach ambient temperature and Na_2_CO_3_ (630 mg, 5.95 mmol) was added. Mixture was stirred for 1 h then a solution of BOC_2_O (1 g, 4.58 mmol) in CH_2_Cl_2_ (20 ml) was slowly added. The reaction proceeded under stirring overnight, then it was filtered ad washed with 10% NaHCO_3_ solution (3 × 20 ml) and with water (2 × 20 ml). Organic phase was dried over sodium sulphate; solvent was removed under reduced pressure and compound (b) was collected in 87.5% yield (1.13 g).

^1^H NMR (300 MHz, CDCl_3_, *δ* 7.26 ppm) *δ* 7.19 (s, 5H), 4.94 (s, 2H + 2H), 3.99 (sb, 1H), 3.02 (sb, 2H), 1.73–0.96 (m, 9H + 9H + 6H); ^13^C NMR (75 MHz, CDCl3, *δ* 77.2 ppm) *δ* 171.8, 156.5, 155.5, 146.7, 128.4, 128.0, 127.9, 85.0, 81.6, 79.5, 66.4, 53.7, 32.42, 31.1, 28.3, 27.9, 22.3; ESI-MS^+^: *m*/*z* calcd for C_23_H_36_N_2_O_6_, 436.26; found 459.20 [M + Na^+^].

#### Synthesis of compound (c) (LysBoc-*t*Bu): *tert*-butyl 6-amino-2-((*tert*-butoxycarbonyl)-amino) hexanoate

Compound (b) (5 g, 11.5 mmol) was dissolved in methanol (100 ml) and Pd/C (10%, 500 mg) was added. The mixture was stirred at room temperature under hydrogen atmosphere (atmospheric pressure) for 48 h. Then, it was filtered and solvent was removed under reduced pressure obtaining a white solid (2.4 g). Acidimetric titration indicates that this solid was formed by compound (c) (40%) and its carbamic acid form (60%). The solid was dissolved in methanol, cooled down to 5 °C in an ice bath, and aqueous HCl (1 M, 4.6 ml) was slowly added. The CO_2_ was removed under vacuum, NaOH (2 M) was added until pH = 9, methanol was evaporated under reduced pressure ad resulting aqueous phase was extracted with ethyl acetate (3× 50 ml). Organic layer was dried over sodium sulphate and solvent evaporated to obtain compound (c) in 15% yield. Acidimetric titration indicates 85% of purity.

^1^H NMR (300 MHz, CDCl_3_, *δ* 7.26 ppm) §§Some impurities were observed; however chemical shifts refer to most abundant compound.*δ* 3.82 (sb, 1H), 2.48 (sb, 2H), 1.82–0.70 (overlapped, 6H + 18H); ESI-MS^+^: *m*/*z* calcd for C_15_H_30_N_2_O_4_, 302.22; found 303.19 [M + H^+^].

#### Synthesis of compound (2) *meso*-tetrakys-(4-(5-amino-5-carboxypentylaminocarbonyl)-phenyl)-porphyrin

Commercial *meso*-tetrakys-(4-carboxyphenyl)-porphyrin (64 mg, 0.08 mmol) was dissolved in a mixture of CH_2_Cl_2_ (30 ml) and THF (10 ml), then EDC (165 mg, 0.86 mmol) and DMAP (52 mg, 0.43 mmol) were added. The mixture was stirred at RT for 30 min and a solution of compound (c) (306 mg, 85% purity, 0.86 mmol) in CH_2_Cl_2_ (30 ml) was slowly dropped. The reaction progress was monitored by TLC (silica, CH_2_Cl_2_/MeOH = 9/1; *R*_f_ = 0.87), HPLC (Waters SunFire C18 column −4.6 × 150, 5 μm; flow = 1 ml min^−1^; eluent A = 0.1% TFA in water, B = 0.1% TFA in MeOH, gradient – time, B%- 0, 75; 15, 75; 27.5, 100; 42.4, 100; *R*_t_ = 29.05 min) and ESI-MS. After 4 h the reaction was completed and the mixture was separated by flash chromatography (Silica cartridge 12 g, CH_2_Cl_2_/MeOH: column volume, MeOH%- 0,0; 10,0; 12, 19.6; 15.3, 19.6; 17.3, 39.9; 21.3, 100; 25.3, 100) and the product containing fraction was further purified by preparative HPLC-MS (Waters SunFire C18 column −19 × 100, 5 μm; flow = 20 ml min^−1^; eluent A = water, B = MeOH, gradient – time, B%- 0, 75; 12.5, 100; 16, 100) obtaining 50 mg of (2) in 34% yield. The ^1^H-NMR spectrum shows the presence of (2) in mixture with its partially deprotected derivatives which do not compromise its use in the next deprotection step. The solid was then dissolved in CH_2_Cl_2_ (20 ml) and TFA (3 ml) was slowly added. The mixture was stirred overnight and solvent and excess of TFA were evaporated under reduced pressure obtaining a blue/green solid (34 mg, yield 53%).

^1^H NMR (300 MHz, CD_3_OD, *δ* 3.31 ppm) *δ* 8.82 (s, 8H), 8.31 (m, 16H), 4.07 (sb, 4H), 3.62 (sb, 8H), 2.06 (sb, 8H), 2.29–1.5 (overlapped, 24H); ^13^C NMR (75 MHz, CD_3_OD, *δ* 49.0 ppm, from HSQC) *δ* 136.8, 131.4, 127.2, 53.5, 40.4, 30.8, 29.9, 23.2; ESI-MS^+^: *m*/*z* calcd for C_72_H_78_N_12_O_12_, 1302.59; found 1304.0 [M + H^+^], 652.6 [M + 2H^+^].

#### Synthesis of compound (3)

Compound (3) was synthesized accordingly to previously described procedure,^30^ developed by authors, in 46%. Yield (28.5 mg) ^1^H NMR (300 MHz, CD3OD, *δ* 3.31 ppm) *δ* 8.50 (s, 8H), 8.28 (br. t, 4H), 7.96 (br. d, 4H), 7.36 (d, *J* = 5.64 Hz, 8H), 7.31 (br. d, 4H) 6.33 (d, *J* = 5.84 Hz, 8H), 3.66–3.54 (br. m, 16H + 16H + 8H), 3.07 (br. s, 8H).

#### Synthesis of SWCNT–1

SWCNT (20 mg), compound 1 (40 mg, 0.07 mmol) and DMF (4 ml) were placed in a specific glass tube (Anton Parr G10). The tube was placed in the MW oven, sealed and heated at 100 °C (temperature-controlled MW irradiation with max power of 600 W) for 2 h. The mixture was then cooled to RT, centrifuged and washed with DMF (3 ml) and CH_2_Cl_2_ (3× 4 ml). The black solid was recovered and dried at 100 °C for 48 h (21 mg). The final product was characterized by Raman spectroscopy, TGA, AFM and TEM.

#### Synthesis of SWCNT–2

SWCNT (30 mg), octanal (26 mg, 32 μl, 0.20 mmol), compound 2 (55 mg, 0.04 mmol) and DMF (3 ml) were placed in a specific glass tube (Anton Parr G10). The tube was placed in the MW oven, sealed and heated at 120 °C (temperature-controlled MW irradiation with max power of 600 W) for 2 h. The mixture was then cooled to RT, centrifuged and washed with DMF (3 ml) and CH_2_Cl_2_ (3× 4 ml). The solid was recovered and dried at 100 °C for 48 h. The final product was characterized by Raman spectroscopy and TGA.

#### Synthesis of SWCNT–3

SWCNT (10 mg), compound 3 (10 mg, 6 μmol) and DMF (3 ml) were placed in a specific glass tube (Anton Parr G10). The tube was placed in the MW oven, sealed and heated at 100 °C (temperature-controlled MW irradiation with max power of 600 W) for 2 h. The mixture was then cooled to RT, centrifuged and washed with DMF (3 ml) and CH_2_Cl_2_ (3× 4 ml). The black solid was recovered and dried at 100 °C for 48 h (14 mg). The final product was characterized by Raman spectroscopy and TGA.

### Preparation of PEG–SWCNTs

SWCNT (1 mg) were suspended in 2 ml of mPEG-DSPE solution (2.5 mg ml^−1^ in PBS pH 7.4) and bath sonicated for 3 h. Finally, unbound conjugate was removed by repeat filtering and washing with PBS (Centrisart® I centrifugal ultrafiltration unit NMWCO 100 kDa, Sigma-Aldrich).

1 mg of the different PEG-SWCNTs were added to 2 ml of different medium [water, PBS, RPMI (Roswell Park Memorial Institute medium) 1640 and RPMI 1640 + 10% FBS (foetal bovine serum)] and bath sonicated for 1 h then stability within 30 days was evaluated. The suspension appears stable after so long period (Fig. 5,† in ESI,† shows the porphyrin–CNT–PEG suspension after 30 days from its preparation).

For the *in vitro* experiments PEG–SWCNTs were suspended in PBS containing 10% percoll® at a concentration of 500 μg ml^−1^.

### Cell culture and treatments

Human colorectal cancer cell line, HT-29 (ICLC, Interlab Cell Line Collection, Genova, Italy), was cultured in RPMI-1640 medium, supplemented with 2 mM l-glutamine, 100 IU ml^−1^ penicillin, 100 μg ml^−1^ streptomycin and 10% (v/v) heat-inactivated fetal calf serum (Sigma-Aldrich) in a humidified atmosphere of 5% CO_2_ air at 37 °C.

Cells in the exponential growth phase were harvested with 0.25% trypsin and normalized to 5.0 × 105 cells in 3 ml of medium containing 10% of percoll® and 25 μg ml^−1^ of SWCNT and porphyrin-loaded SWCNT (SWCNT–1–PEG, SWCNT–2–PEG and SWCNT–3–PEG) in polystyrene tubes for light beam (LB) or US exposure.

LB exposure was carried on by custom system, adjusting energy fluency rates of light radiation at 15 mW cm^−2^, for 5 min. The light intensity was measured using Actinic UV-meter (Jelosil, Le Landeron, CH).

US cells irradiation was carried out at *f*_0_ = 1.866 MHz and with an energy density corresponding to 0.008 mJ cm^−2^; energy supplied to the cells did not increase the medium temperature (maximum temperature recorded was 33 °C).

### Cell proliferation assay

The effect that porphyrin loaded SWCNT had on HT-29 cell proliferation was evaluated by WST-1 cell proliferation assay (Roche Applied Science, Penzberg, Germany). After various treatments, 2.0 × 10^3^ HT-29 cells were seeded in 100 μl of growth medium in replicates (*n* = 8) in 96-well culture plates (TPP, Trasadingen, Switzerland). At 24 and 48 h, WST-1 reagent (10 μl) was added and the plates were incubated at 37 °C in 5% CO_2_ for 1.5 h. Well absorbance was measured at 450 and 620 nm (reference wavelength) on a microplate reader Asys UV 340.

### Statistical analyses

Data are shown as average values ± standard deviation of three independent experiments. Statistical analyses were performed on Graph-Pad Prism 6.0 software (La Jolla, CA, USA); two-way analysis of variance and Bonferroni's test were used to calculate the threshold of significance. Statistical significance was set at *p* < 0.05.

### Reactive oxygen species (ROS) generation/terephthalate assay

Terephthalate (TA) solution (8 mM) was prepared dissolving disodium terephthalate in Milli-Q ultrapure water. A stable SWCNT-1 suspension was obtained suspending SWCNT–1 in DMSO (purity 99.9%). TA solution was added to the DMSO : water (1 : 3) suspension of SWCNT–1. The concentration of TA and SWCNT–1 in the final mixture was 4.0 mM, and 25 μg ml^−1^, respectively. The suspension was introduced in a polystyrene tube (volume = 3 ml; tube diameter = 1 cm) and exposed to US (1.866 MHz) under the same experimental conditions employed in cell experiments (namely 0.008 mJ cm^−2^ for 5 min). Sonication was carried out using also different sonotrode frequency (22 kHz): 2 ml of suspension were introduced in polystyrene tubes and exposed to ultrasound (10 kJ cm^−2^ for 1 min) employing a sonicator Sonopuls HD3100 equipped with a 73 MS probe (Bandelin, Germany). After the US treatment, the suspension was filtered on a cellulose acetate membrane (pore diameter 0.45 μm, Advantec, Japan) and TA-OH fluorescence (*λ*_ex_ = 324 nm, *λ*_em_ = *ca.* 425 nm) was measured on the clear solution by a FLx800 fluorescence reader (BioTek, US). The results were compared to a blank solution without US irradiation and on US-irradiated pristine SWCNT (25 μg ml^−1^) under the same experimental conditions. Three replicates for each experiment were carried out and the results were expressed as the mean values ± SD of the separate determinations and analysed by a one-way Analysis of Variance (ANOVA) and Tukey's test. *p* < 0.05 was considered significant.

### Physico-chemical characterization of porphyrin-conjugated SWCNTs

To assess the structural and morphological modifications possibly induced by conjugation on SWCNT, several physico-chemical characterization techniques were complementary used. Raman spectroscopy (Fig. 5), thermogravimetric analysis (Fig. S7 and S8†), and electron and atomic force (Fig. S9†) microscopy were carried out on representative samples from each synthetic route. Namely, the SWCNT conjugated *via* Diels–Alder cycloaddition (compounds SWCNT–1 and SWCNT–3) and azomethine ylide 1,3-dipolar cycloaddition (SWCNT–2) were thoroughly characterized comparing pristine SWCNT.

## Conclusions

In this study, we propose a novel covalently grafted SWCNT–porphyrin hybrid nanosystem, which is biologically active only upon US irradiation thanks to the synergistic action of the two bonded entities. The hybrid nanosystem is non-toxic *per se in vitro* but kills up to 75% HT-29 colon cancer cell under low-power US irradiation. The selectivity (no effect under LB) and high efficiency (40-fold higher than PCNP) of these hybrid nanosystems are here demonstrated for the first time and it might be due to the increase of the separated-charge state lifetime, which promotes ROS production and, in turn, may impair cell proliferation. The lack of photo-induced effects of these SWCNT–porphyrin could allow to overcome the big limit of skin photosensitivity. The here described hybrid nanosystem represents an interesting model for a further development of selective US-activated anticancer sonosensitizer.

## Conflicts of interest

There are no conflicts to declare.

## Supplementary Material

RA-010-D0RA03944F-s001
