# Supplementary material for: SWCNT–porphyrin nano-hybrids selectively activated by ultrasound: an interesting model for sonodynamic applications
Source: RSC Adv. 2020 Jun 8;10(37):21736–44. doi: 10.1039/d0ra03944f (PMC9054547; doi:10.1039/d0ra03944f)
Supplement: RA-010-D0RA03944F-s001 [file RA-010-D0RA03944F-s001.pdf]

Electronic Supplementary Material (ESI) for RSC Advances.
This journal is © The Royal Society of Chemistry 2020

Electronic Supporting Information

SWCNT-porphyrin nano-hybrids selectively activated by ultrasound: an interesting model for sonodynamic applications

Federica Bosca,[a‡] Ingrid Corazzari,[b‡] Federica Foglietta, [a] Roberto Canaparo, [a] Gianni Durando,[c] Linda pastero,[d,e] Silvia Arpicco, [a] Franco Dosio, [a] Daniele Zonari, [a] Giancarlo Cravotto, [a] Silvia Tagliapietra, [a] Loredana Serpe, [a] Francesco Turci [b,e] and Alessandro Barge,*[a]

a) Department of Drug Science and Technology, University of Turin, Turin, Italy.
b) Department of Chemistry, University of Turin, Turin, Italy.
c) National Institute of Metrological Research (INRIM), Turin, Italy.
d) Department of Earth Sciences, University of Turin, Turin, Italy
e) "G. Scansetti" Interdepartmental Centre, University of Turin Via P. Giuria 9, 10125 Turin, Italy

‡ first author

Contents

Scheme S1. Synthetic procedure to obtain suitable protected lysine (c). 2
Scheme S2. Lysin conjugation to commercial porphyrin. 3
Scheme S3. Structures of synthesized porphyrins 3
Figure S1. ^{1}H, ^{13}C spectra of compound a. 4
Figure S2. ^{1}H, ^{13}C spectra of compound b. 5
Figure S3. ^{1}H spectrum of compound c. 6
Figure S4. ^{1}H, HSQC spectra of compound 2. 7
Figure S5. Suspension stability of SWCNT (top) and SWCNT-PEG (bottom) in different media at 30 days after preparation 8
Figure S6. TGA profiles of SWCNT (black) and SWCNT-PEG (red) 8
Figure S7. Thermograms recorded with SWCNT-1, SWCNT-2, SWCNT-3 in the temperature range between 25 and 700 °C (heating rate 20°C min^{-1}) under dynamic nitrogen atmosphere (purity 99.9995%; flow 35 ml min-1). The thermogram with pristine SWCNT is reported for comparison. 10
Figure S8. (A) Representative FTIR spectrum of the gas evolved at 210 °C from SWCNT-2 (curve a) compared with the FTIR spectrum of DMF from Perkin Elmer Spectrum database (curve b); (B) FTIR profiles of DMF release from SWCNT-1 (a'), SWCNT-2 (b') and SWCNT-3 (c') obtained expressing the absorbance measured at 1720 cm^{-1} as a function of the temperature. 10
Figure S9. AFM images of SWCNT and SWCNT-1 (a and b respectively) and topographic profiles (each height profile corresponds to an arrow in the AFM image). In all samples, the ribbons' width ranges from 150 nm to 500 nm and their height ranges from 5 nm to 15 nm (a1, a2 and b1, b2 and b3). In the SWCNT sample, some smaller features (approximately 150 nm large and 1.5 nm high) are recognized (a3). 11

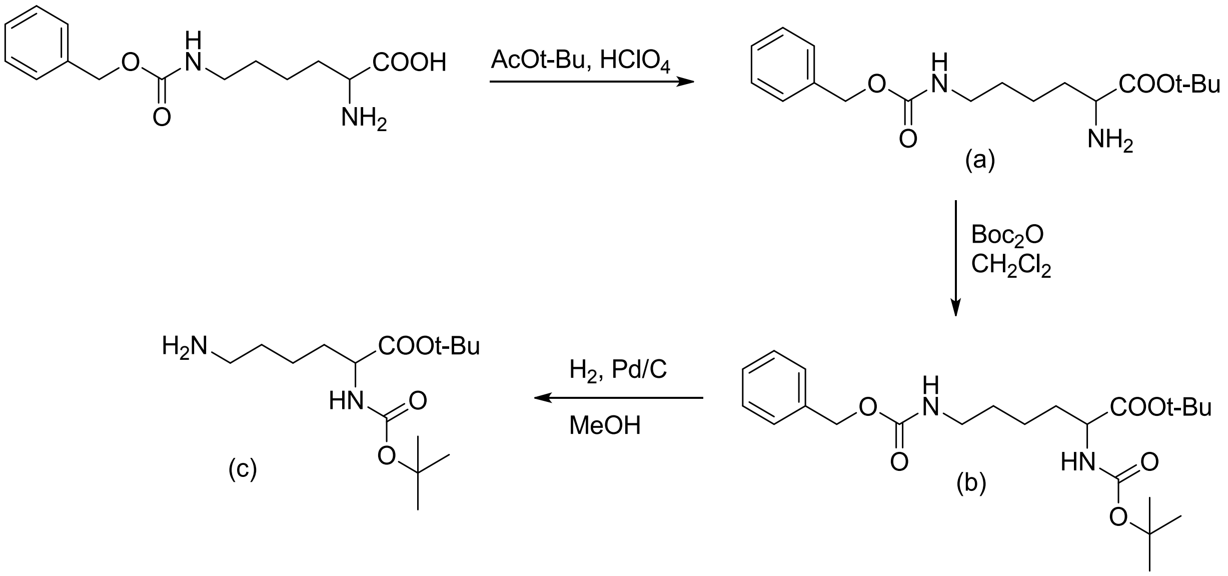

Scheme S1. Synthetic procedure to obtain suitable protected lysine (c).

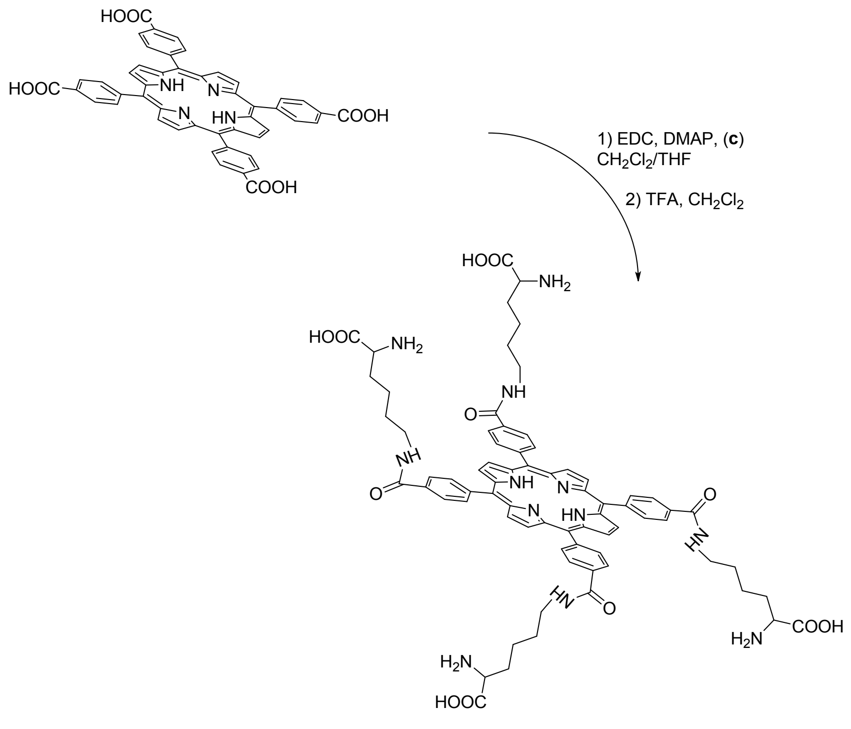

Scheme S2. Lysin conjugation to commercial porphyrin.

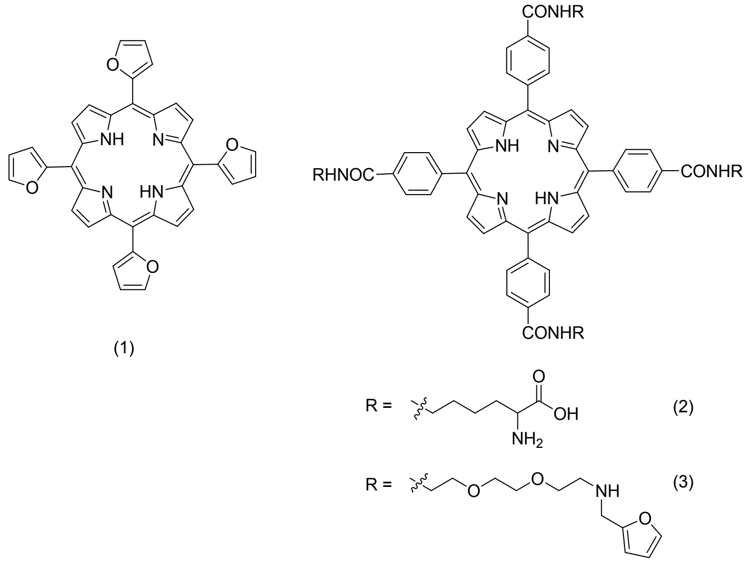

Scheme S3. Structures of synthesized porphyrins

NMR Spectra

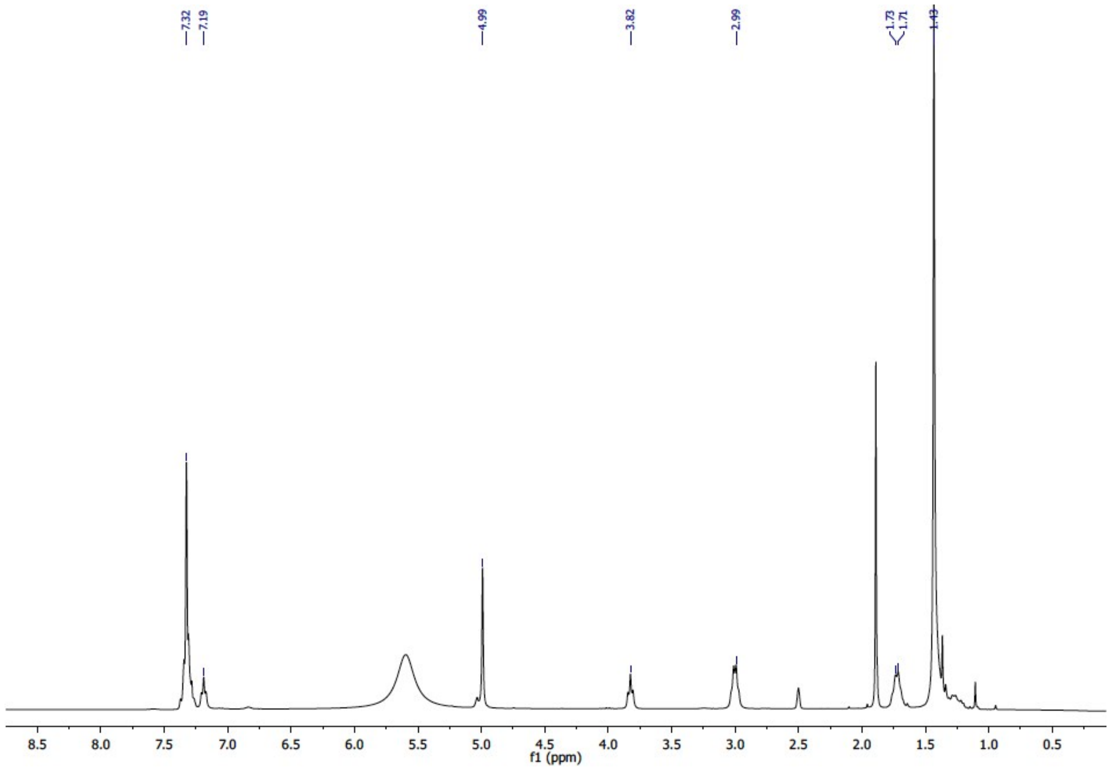

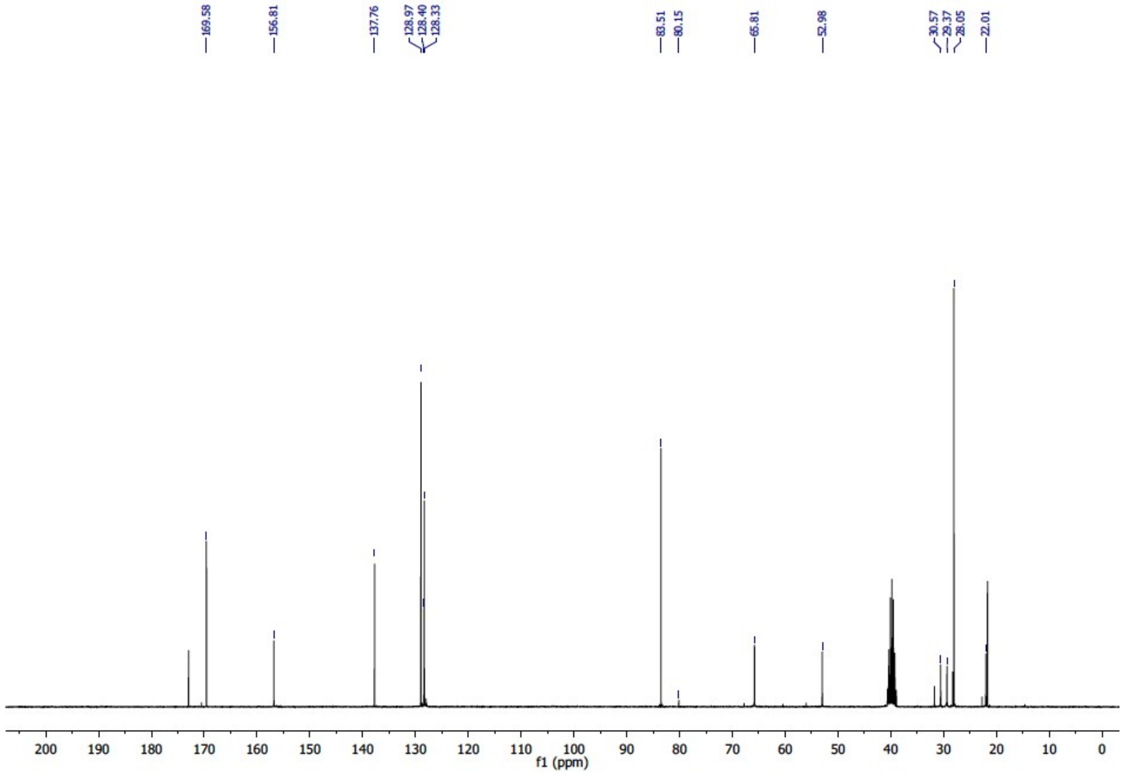

Figure S1. ^{1}H, ^{13}C spectra of compound a.

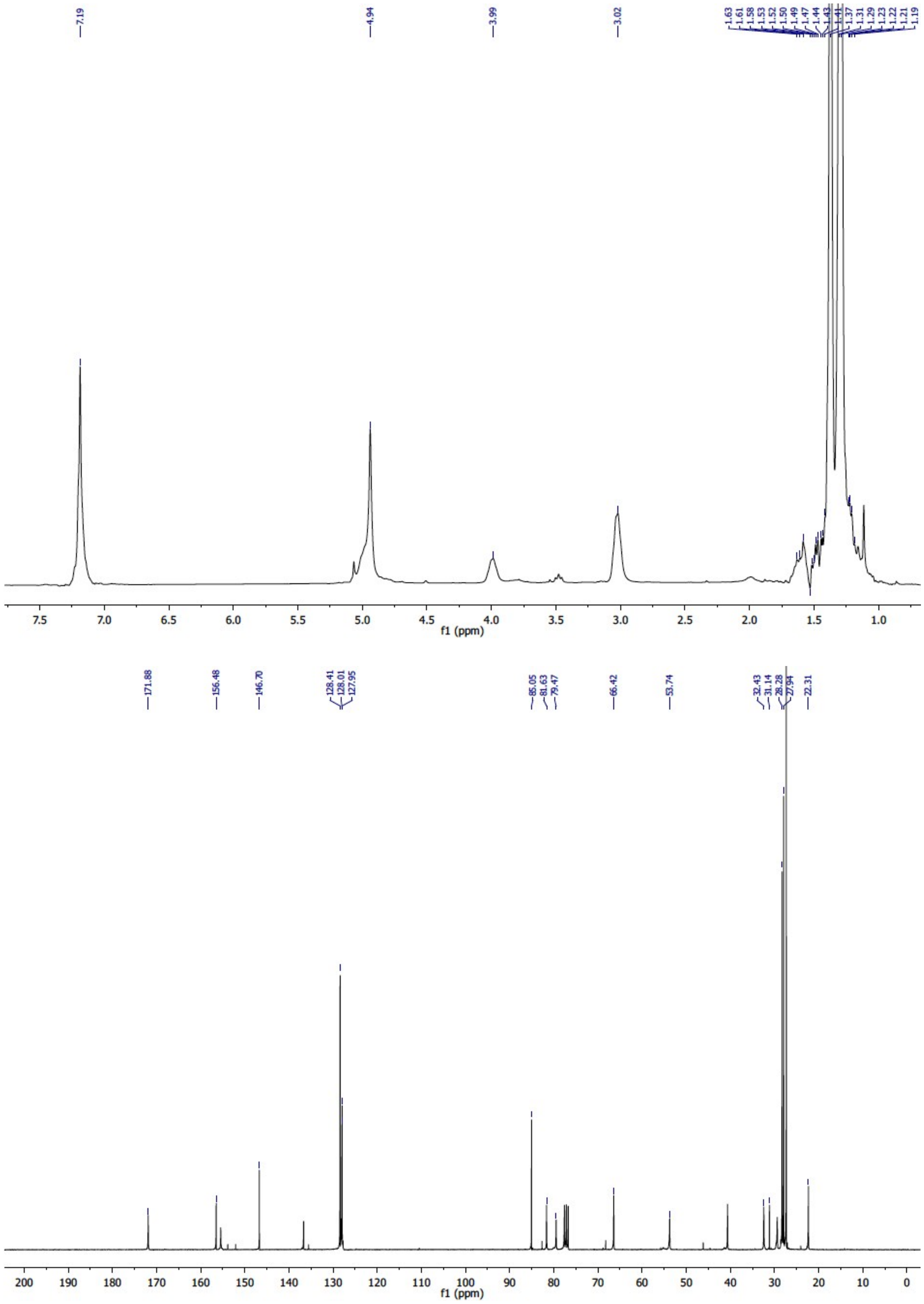

Figure S2. ^{1}H, ^{13}C spectra of compound b.

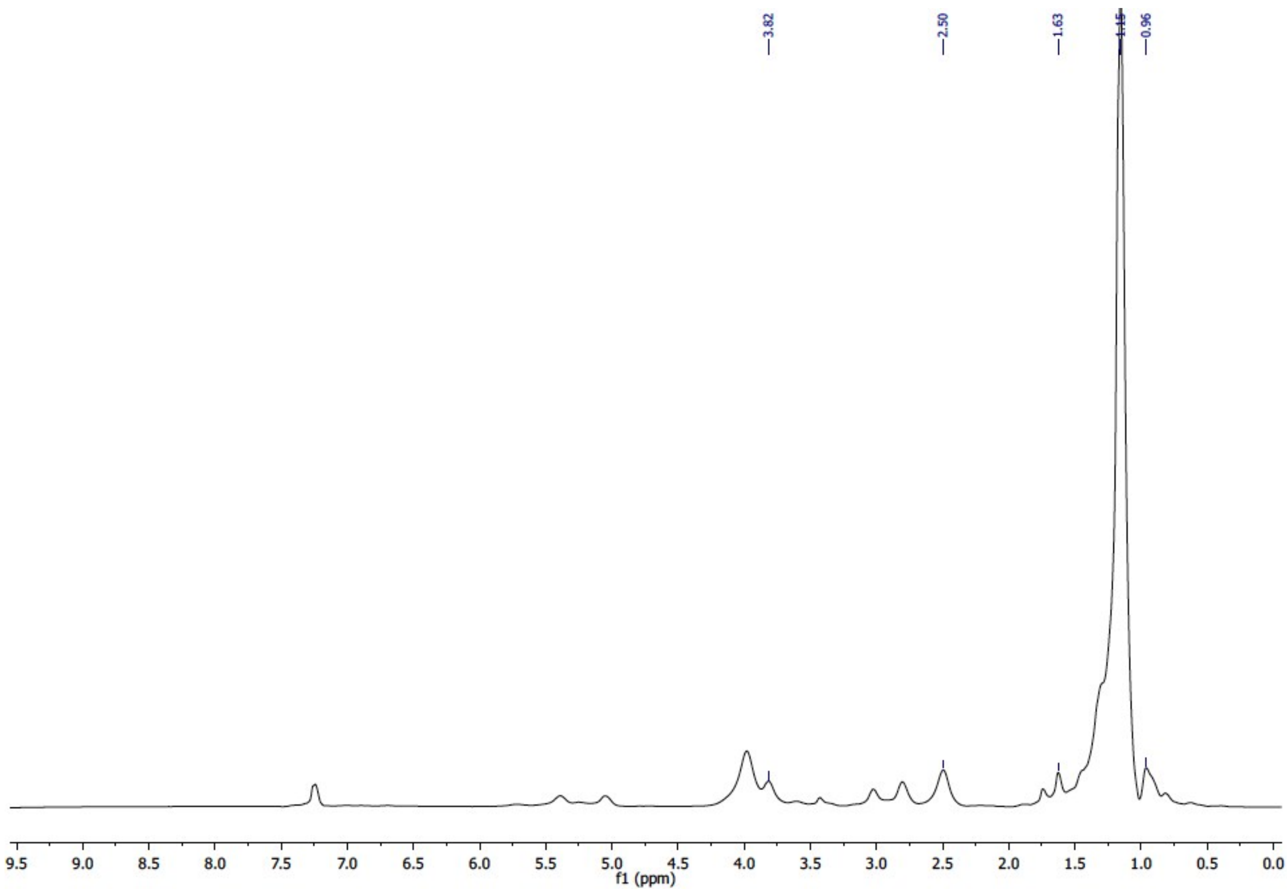

Figure S3. ^{1}H spectrum of compound c.

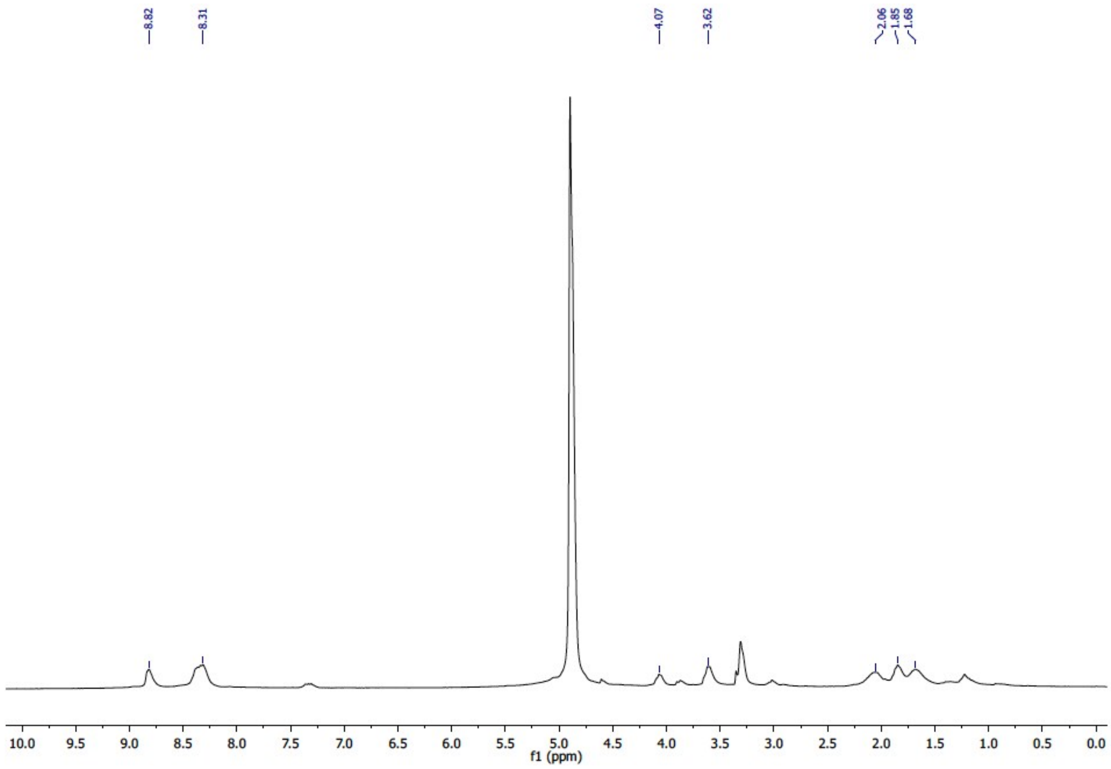

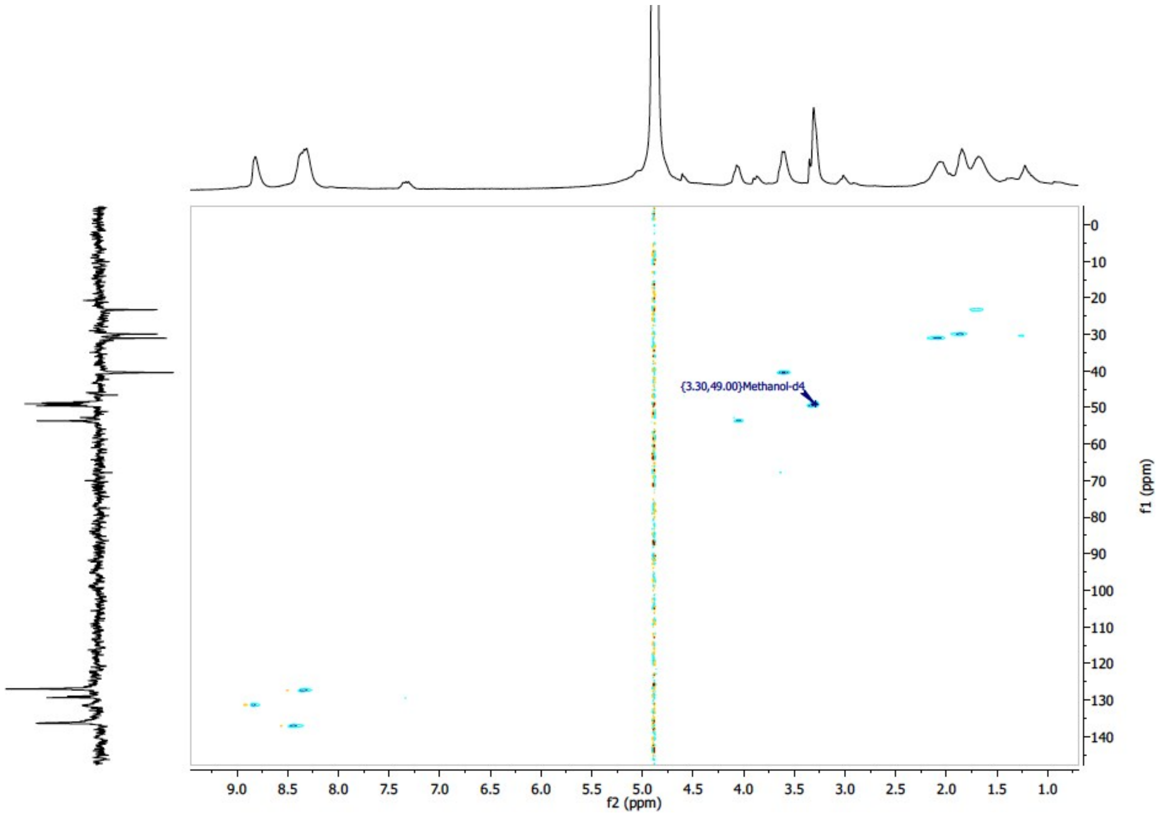

Figure S4. ^{1}H, HSQC spectra of compound 2.

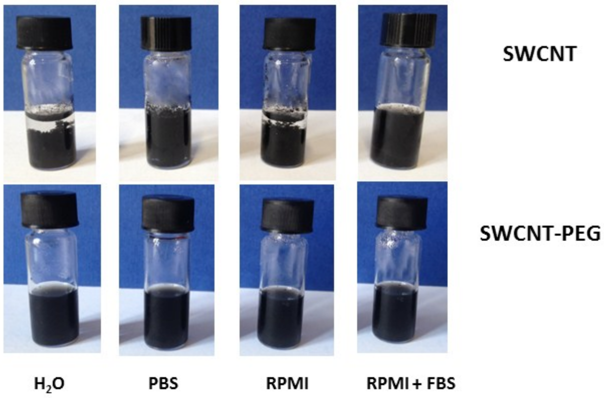

Figure S5. Suspension stability of SWCNT (top) and SWCNT-PEG (bottom) in different media at 30 days after preparation

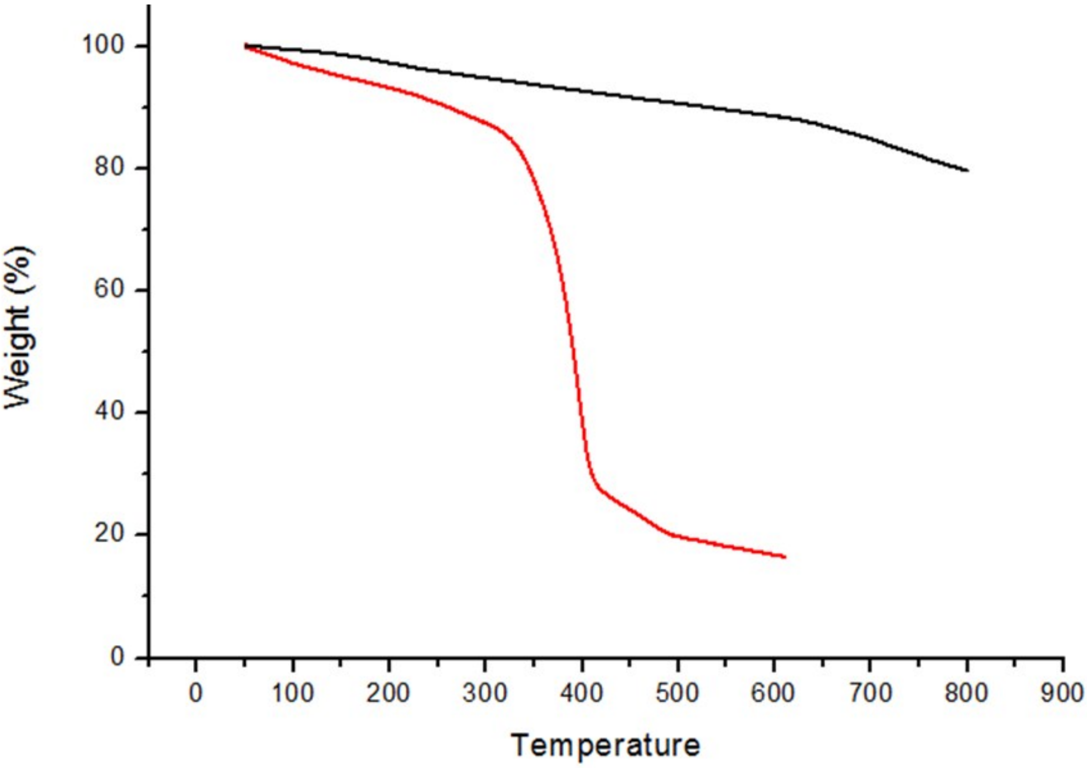

Figure S6. TGA profiles of SWCNT (black) and SWCNT-PEG (red)

Functionalization degree of hybrid conjugates

To assess the degree of functionalization, the thermal behavior of SWCNT-1, SWCNT-2 and SWCNT-3 in comparison with the pristine one (SWCNT) was investigated in the temperature range between 25 and 700 °C under inert atmosphere (N_2, purity: 99.9995%, flux rate: 35 ml min^{-1}). To identify the nature of the species evolved, the gas released during the heating was continuously monitored by FTIR. In figure S7 the thermograms are reported. SWCNT-1, SWCNT-2, and SWCNT-3 exhibited an overall mass loss of ca. 10.8% w/w, 12.1% w/w, and 10.2% w/w, which was significantly higher than that observed with the pristine SWCNT (ca. 3.5% w/w). The thermogram was divided into three regions of temperature, namely 25-160 °C (region I), 160-270 °C (region II),

and 270-600 °C (region III). In region I (25-160 °C), the thermal behavior of all the samples analyzed was similar and the weight loss recorded was 1.0% w/w for SWCNT-1, 0.70% w/w for SWCNT-2, and 0.90% w/w for both SWCNT-3 and pristine SWCNT. FTIR analysis clarified the nature of the gas evolved and the weight loss was attributed to the evolution of small amounts of physisorbed water (data not shown for brevity). In region II (160-270 °C), all the functionalized samples exhibited a significant additional weight losses in comparison with the pristine SWCNT (namely 1.4% w/w with SWCNT-1, 0.8% w/w with SWCNT-2 and 0.5% w/w with SWCNT-3). The FTIR spectra recorded allowed to assign such weight loss to the desorption of small amounts of N,N-dimethylformamide (DMF), which is present on the functionalized samples as an inevitable residue deriving from the synthesis. In figure S7 a representative spectrum of the gas evolved from SWCNT-2 is reported (curve a) and compared with the FTIR spectrum of DMF from Perkin Elmer database (curve b). The FTIR profile of the DMF release from the functionalized samples (figure S7 B) evidenced that such species was evolved until ca. 270 °C. At higher temperature (region III, from 271 °C to 600 °C), the thermal behavior of the functionalized samples differed further from that of the pristine SWCNT. Even though the FTIR analysis did not unveil the nature of the gaseous moieties evolved, this result was univocally ascribed to the thermal degradation of porphyrin. The amount of porphyrin was quantified by the additional weight loss in comparison with the pristine SWCNT. The mass attributed to the porphyrin introduced by functionalization process in SWCNT-1, SWCNT-2 and SWCNT-3 was 6.7%% w/w, 8.0 % w/w and 7.0 % w/w, respectively. The molar mass of porphyrins 1, 2 and 3 is 574 g mol^{-1}, 1411 g mol^{-1}, and 1634 g mol^{-1}, respectively. Expressing the amount of porphyrin on a molar basis (µmol of porphyrin x mg^{-1} of functionalized SWCNT), the functionalization degree of SWCNT-1, SWCNT-2 and SWCNT-3 was $1.2x10^{-1}$ µmol mg^{-1}, $5.7x10^{-2}$µmol mg^{-1} and 4.3 $x10^{-2}$ µmol mg^{-1}, respectively.

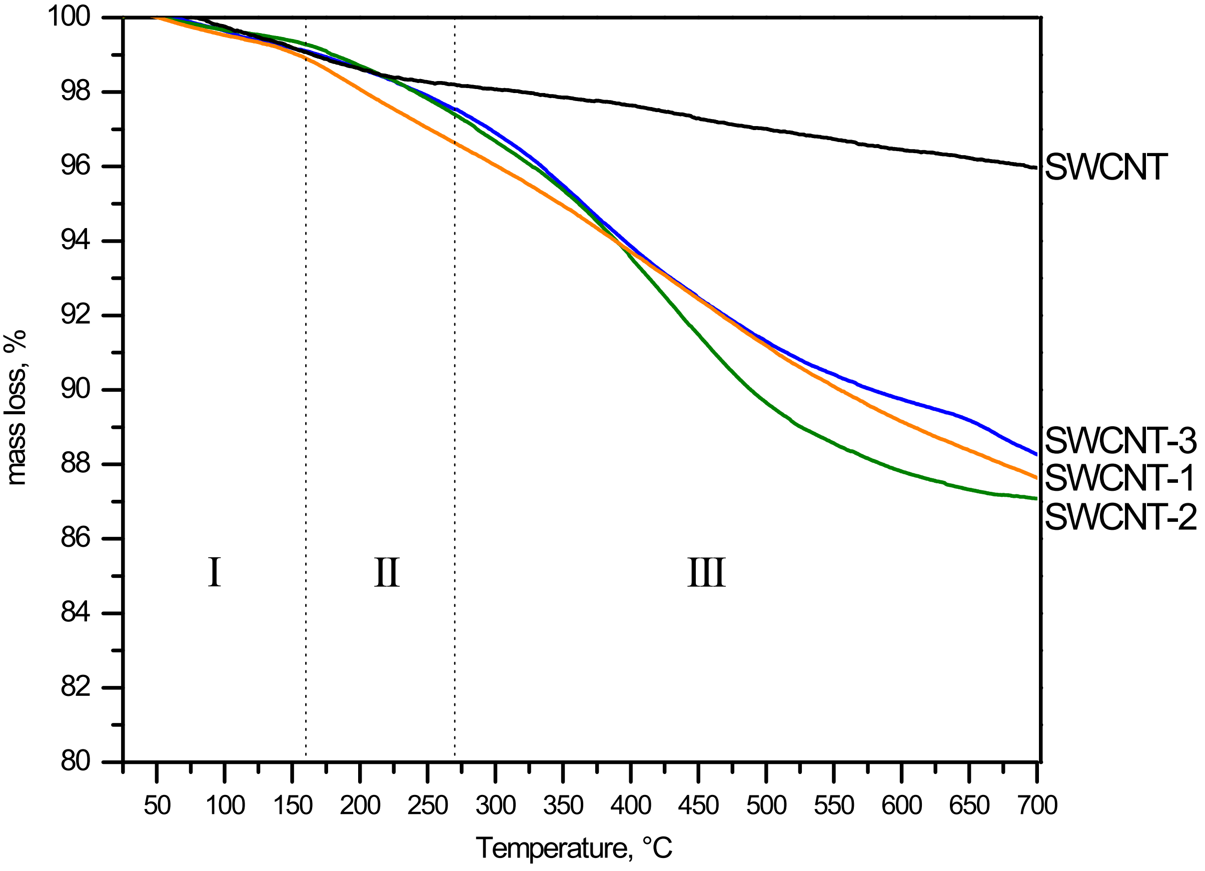

Figure S7. Thermograms recorded with SWCNT-1, SWCNT-2, SWCNT-3 in the temperature range between 25 and 700 °C (heating rate 20°C min^{-1}) under dynamic nitrogen atmosphere (purity 99.9995%; flow 35 ml min-1). The thermogram with pristine SWCNT is reported for comparison.

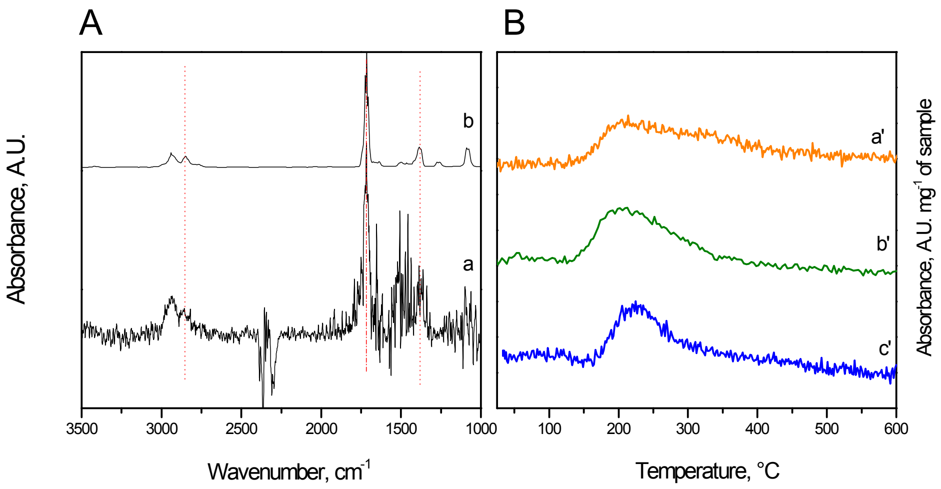

Figure S8. (A) Representative FTIR spectrum of the gas evolved at 210 °C from SWCNT-2 (curve a) compared with the FTIR spectrum of DMF from Perkin Elmer Spectrum database (curve b); (B) FTIR profiles of DMF release from SWCNT-1 (a'), SWCNT-2 (b') and SWCNT-3 (c') obtained expressing the absorbance measured at 1720 cm^{-1} as a function of the temperature.

Height profiles of SWCNT by Atomic force microscopy (AFM)

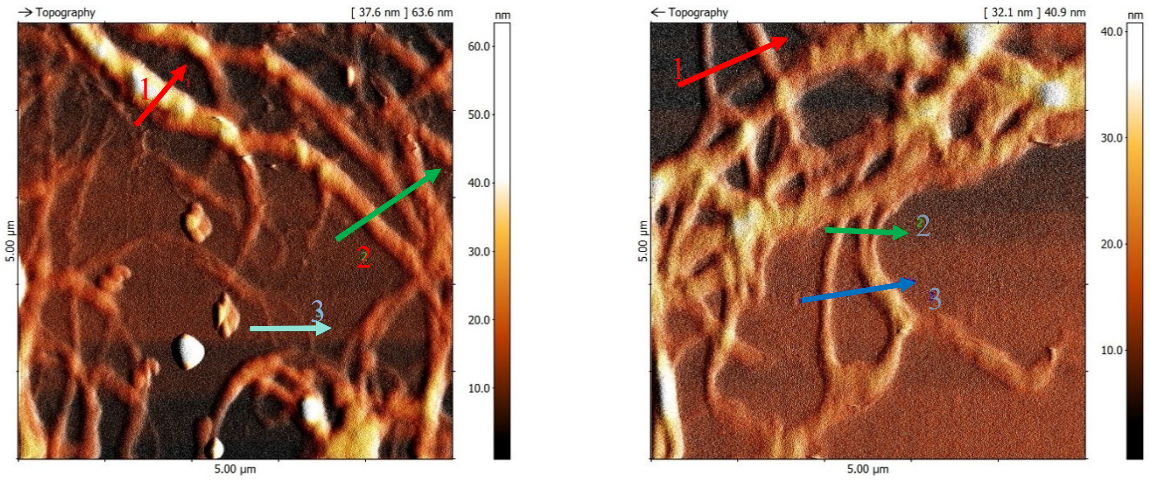

b1

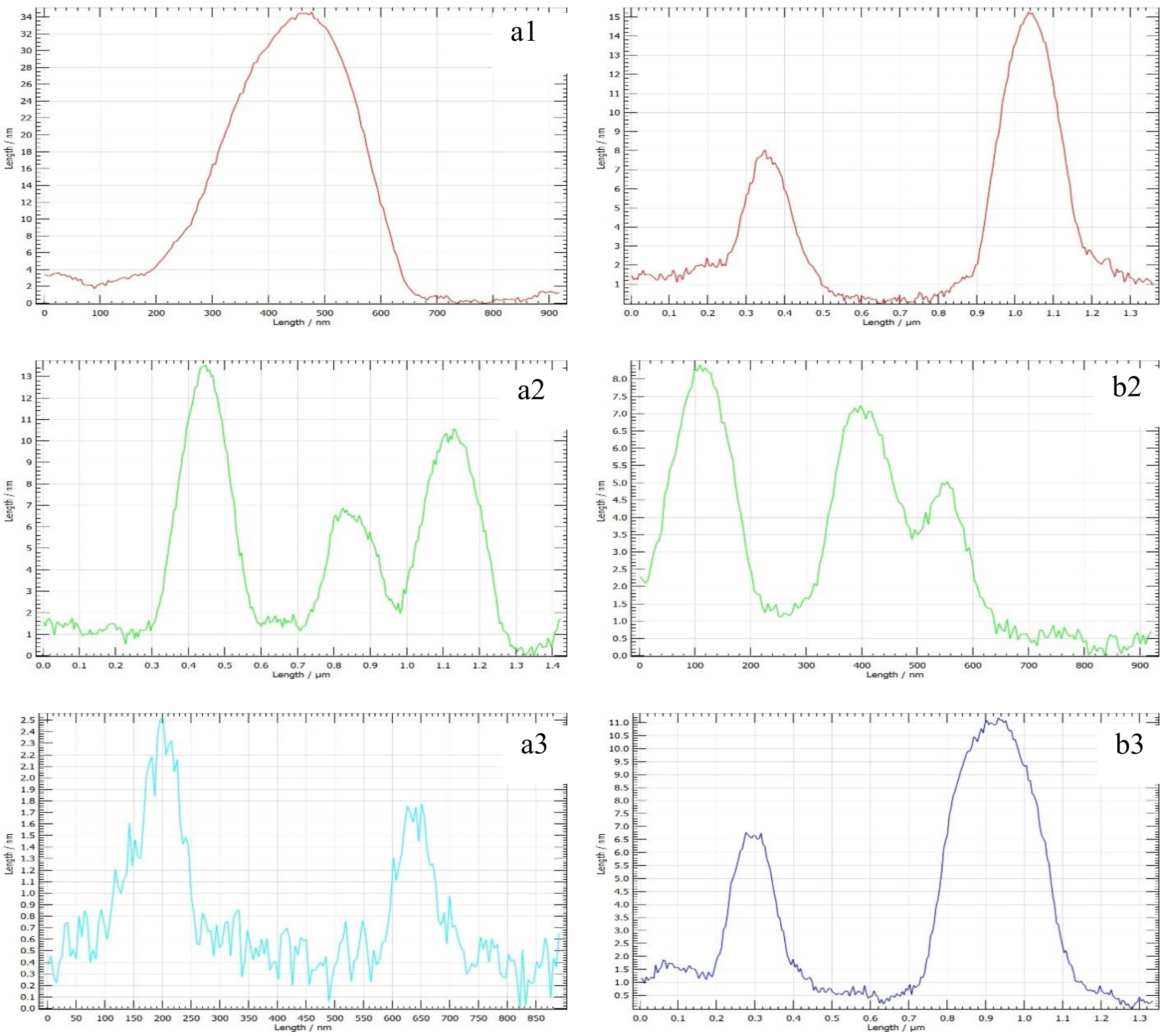

Figure S9. AFM images of SWCNT and SWCNT-1 (a and b respectively) and topographic profiles (each height profile corresponds to an arrow in the AFM image). In all samples, the ribbons' width ranges from 150 nm to 500 nm and their height ranges from 5 nm to 15 nm (a1, a2 and b1, b2 and b3). In the SWCNT sample, some smaller features (approximately 150 nm large and 1.5 nm high) are recognized (a3).
